# Electrolyte Concentration Regulation Boosting Zinc Storage Stability of High-Capacity K_0.486_V_2_O_5_ Cathode for Bendable Quasi-Solid-State Zinc Ion Batteries

**DOI:** 10.1007/s40820-020-00554-7

**Published:** 2021-01-04

**Authors:** Linpo Li, Shuailei Liu, Wencong Liu, Deliang Ba, Wenyi Liu, Qiuyue Gui, Yao Chen, Zuoqi Hu, Yuanyuan Li, Jinping Liu

**Affiliations:** 1 School of Optical and Electronic Information, Huazhong University of Science and Technology, Wuhan, 430074 People’s Republic of China; 2 School of Chemistry, Chemical Engineering and Life Science, and State Key Laboratory of Advanced Technology for Materials Synthesis and Processing, Wuhan University of Technology, Wuhan, 430070 People’s Republic of China; 3 Key Laboratory for Photonic and Electronic Bandgap Materials, Ministry of Education, School of Physics and Electronic Engineering, Harbin Normal University, Harbin, 150025 People’s Republic of China

**Keywords:** Electrolyte concentration regulation, Quasi-solid-state Zn ion battery, K_0.486_V_2_O_5_, Large interlayer spacing, Cycling stability

## Abstract

Vanadium-based cathodes have attracted great interest in aqueous zinc ion batteries (AZIBs) due to their large capacities, good rate performance and facile synthesis in large scale. However, their practical application is greatly hampered by vanadium dissolution issue in conventional dilute electrolytes. Herein, taking a new potassium vanadate K_0.486_V_2_O_5_ (KVO) cathode with large interlayer spacing (~ 0.95 nm) and high capacity as an example, we propose that the cycle life of vanadates can be greatly upgraded in AZIBs by regulating the concentration of ZnCl_2_ electrolyte, but with no need to approach “water-in-salt” threshold. With the optimized moderate concentration of 15 m ZnCl_2_ electrolyte, the KVO exhibits the best cycling stability with ~ 95.02% capacity retention after 1400 cycles. We further design a novel sodium carboxymethyl cellulose (CMC)-moderate concentration ZnCl_2_ gel electrolyte with high ionic conductivity of 10.08 mS cm^−1^ for the first time and assemble a quasi-solid-state AZIB. This device is bendable with remarkable energy density (268.2 Wh kg^−1^), excellent stability (97.35% after 2800 cycles), low self-discharge rate, and good environmental (temperature, pressure) suitability, and is capable of powering small electronics. The device also exhibits good electrochemical performance with high KVO mass loading (5 and 10 mg cm^−2^). Our work sheds light on the feasibility of using moderately concentrated electrolyte to address the stability issue of aqueous soluble electrode materials.

**Electronic supplementary material:**

The online version of this article (10.1007/s40820-020-00554-7) contains supplementary material, which is available to authorized users.

## Highlights

- Moderate-concentration ZnCl_2_ (15 m) was found to be effective for suppressing the dissolution of vanadate cathode, which was more stable and had 4 times higher ionic conductivity than 30 m “water-in-salt” electrolyte.
- K_0.486_V_2_O_5_ with huge interlayer space of ~ 0.95 nm was chosen for the first time to assemble aqueous Zn ion batteries, giving rise to excellent rate performance and high energy and power densities.
- A novel sodium carboxymethyl cellulose-15 m ZnCl_2_ hydrogel electrolyte with high ionic conductivity of 10.08 mS cm^−1^ was designed, enabling a bendable Zn ion battery with outstanding resistance to temperature and pressure.

**Electronic supplementary material:**

The online version of this article (10.1007/s40820-020-00554-7) contains supplementary material, which is available to authorized users.

## Graphical abstract

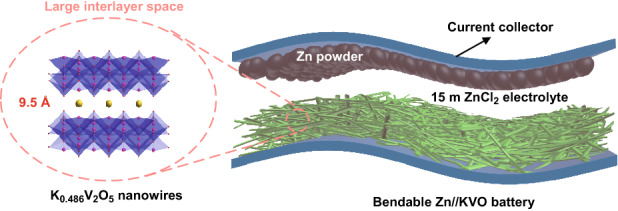

## Introduction

Traditional Li-ion batteries have been considered as the optimal power sources in our daily life owing to their long cycle life and high energy density. Nonetheless, toxicity, safety hazard, and cost issues arising from organic electrolytes have made a big impact on their future development [1–3]. The intrinsic shortcomings motivate researchers to explore more reliable high-energy power supplies. In recent years, aqueous energy storage devices such as aqueous Li^+^, Na^+^, and multivalent ion (Mg^2+^, Ca^2+^, Zn^2+^) batteries have attracted great interest as alternative secondary batteries due to the advantages of low cost, environmental benignity and capability of fast charging. Among them, Li/Na ion batteries generally exhibit relatively low capacity in aqueous electrolytes due to the limited electron-transfer redox reaction in host materials; aqueous Mg and Ca-ion batteries may use high-capacity metal Mg and Ca as anodes, but it is difficult to realize the Mg/Mg^2+^ and Ca/Ca^2+^ redox couples owing to their too negative potentials, at which water will be significantly electrolyzed. By contrast, aqueous zinc ion batteries (AZIBs) are particularly advantageous in terms of theoretical capacity (~ 820 mAh g^−1^), redox potential of Zn/Zn^2+^ (− 0.763 V versus standard hydrogen electrode) and its reversibility [4–13]. However, it is still quite challenging to find reversible and stable Zn storage cathode materials for AZIBs due to the large atom mass and strong electrostatic interaction between divalent Zn^2+^ and host lattice [14–16]. So far, several types of materials including manganese oxides, Prussian blue analogues and vanadium-based compounds (VBCs) have been exploited. Among them, VBCs are promising as the fantastic cathodes because they have both remarkable theoretical capacity and good rate capability [17–20]. Notably, pre-potassiated V_x_O_y_, where cations serve as “pillars” between [V–O] polyhedron layers, can effectively expand its interlayer space and further optimize Zn^2+^ diffusion kinetics [21–25]. In this regard, layered vanadium oxides with larger interlayer spacing are highly desirable. Nevertheless, when used as cathodes in AZIBs, such vanadates are easy to dissolve into water progressively in traditional zinc salt electrolytes, leading to rapid capacity losses and thus ultimate failure of the electrodes [25–27].

To solve the vanadium dissolution issue, the most common strategy is coating protecting layer such as carbon to avoid the direct contact of active materials with electrolyte [25]. In these cases, the thickness of protecting layers should be carefully optimized to not only restrict the dissolution but also maintain efficient ion transport. Apart from electrode modification, electrolyte design is also beneficial to mitigate vanadium dissolution and improve the cycling stability. For instance, H_3_PO_4_ was added to the electrolyte to suppress dissolution of VOPO_4_ · xH_2_O [26]. However, the addition of H_3_PO_4_ may make the electrolyte more acidic and corrode zinc anode. Na_2_SO_4_ was also added into the ZnSO_4_ electrolyte to alter dissolution equilibrium of Na^+^ from NaV_3_O_8_·1.5H_2_O cathode to maintain the electrochemical stability [27], but this method is only effective for specific electrodes. Using 3 m Zn(CF_3_SO_3_)_2_ instead of common ZnSO_4_ electrolyte was demonstrated to be helpful; unfortunately, the price of Zn(CF_3_SO_3_)_2_ is high [28]. Recently, 30 m “water-in-salt” (WIS) electrolyte with low-cost Zn salts was utilized to essentially address the dissolution problem [29], but superhigh salt concentration with high viscosity and poor interfacial wettability often leads to low ion diffusion kinetics, which is not good to the rate performance of AZIBs. In addition, nearly saturated WIS electrolytes are very sensitive to environments and the salts are easy to precipitate at lower temperatures [30, 31]. Based on the above considerations, it is still highly necessary to search for low-cost Zn^2+^ conductive electrolytes with both good chemical stability and the ability to effectively stabilize vanadate cathode.

**Fig. 1:**
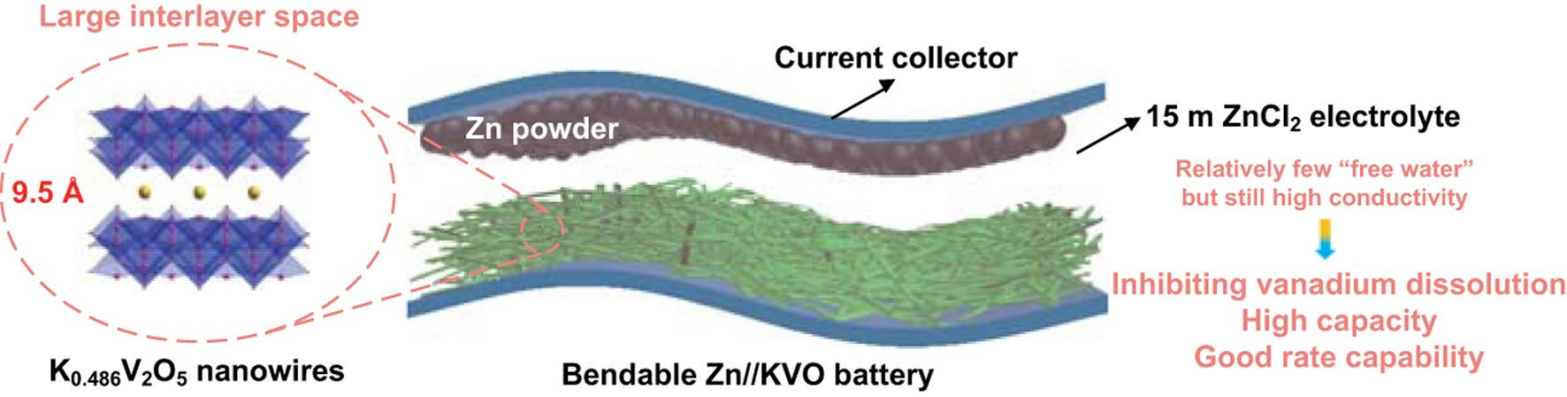
Schematic illustration of bendable Zn//KVO cell with regulating electrolyte concentration

**Fig. 2:**
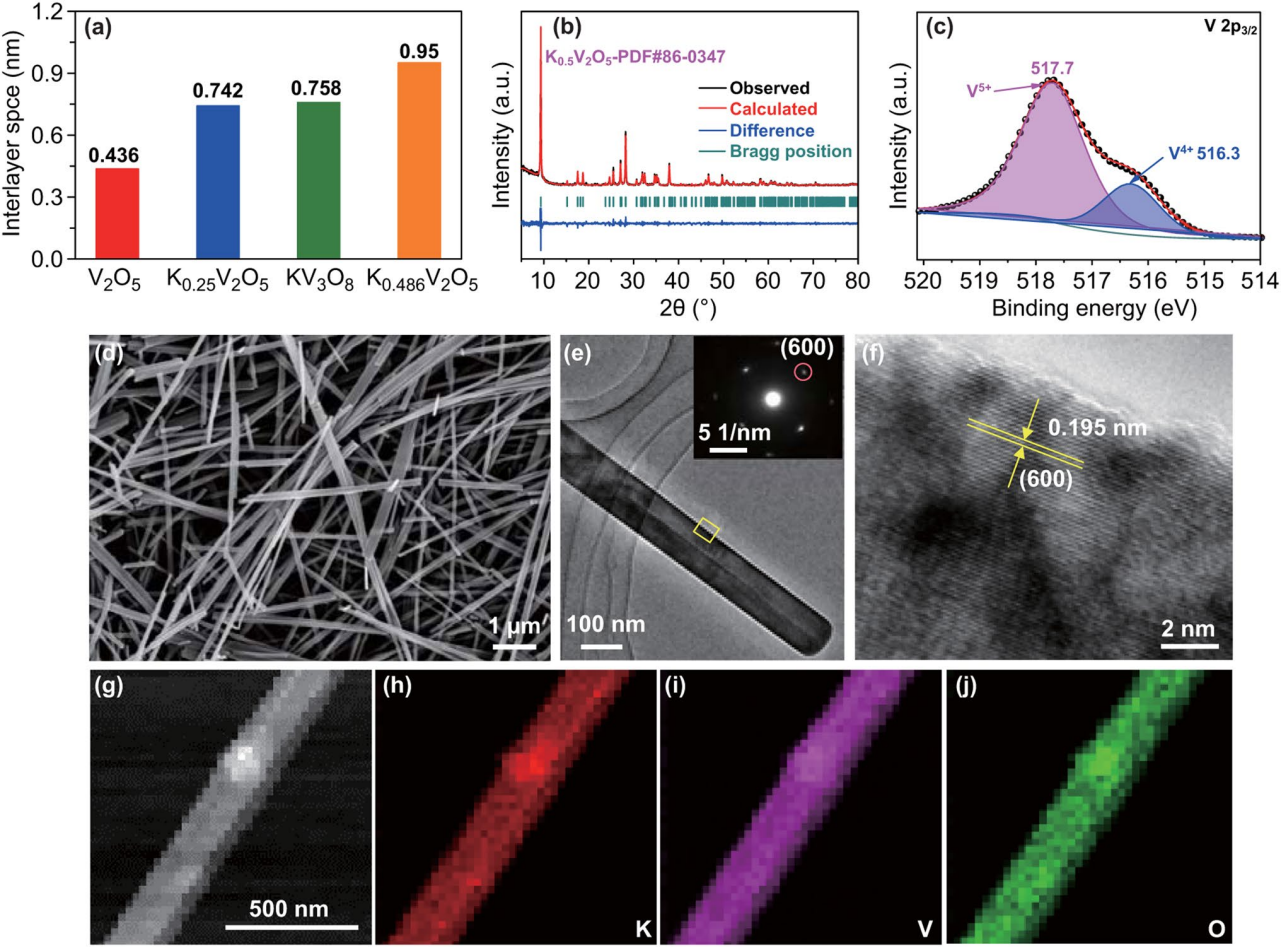
**a** Comparison of interlayer space of various vanadium compounds. **b** XRD pattern of KVO and the corresponding Rietveld refinement results. **c** XPS spectrum of V 2p_3/2_. **d** SEM image, **e** TEM image (inset: SAED pattern), and **f** HRTEM image of KVO. **g–j** EDS mapping

Herein, we discover that a 15 m ZnCl_2_ electrolyte is very effective to prevent the vanadium dissolution of K_0.486_V_2_O_5_ (KVO) while maintaining relatively high ionic conductivity to ensure high capacity and rate capability. We further develop a high-performance AZIB made up of a layered KVO nanowire cathode and a zinc powder anode (Fig. 1). With the purposely designed novel hydrogel electrolyte with moderate-concertation salt, a bendable quasi-solid-state Zn//KVO battery is also assembled. In our AZIB configuration, the main highlights are as follows. (i) Compared with dilute electrolyte, such moderate-concentration ZnCl_2_ greatly suppresses the dissolution of KVO due to the increased viscosity and relatively few “free water”, but compared to WIS Zn^2+^ electrolyte (30 m is the threshold value [32]), it is more stable and has higher ionic conductivity; (ii) it is the first time to use K_0.486_V_2_O_5_ as cathode for Zn storage. The cathode materials have expanded interlayer space of ~ 0.95 nm (Fig. 2a) that is much larger than V_2_O_5_ (~ 0.436 nm), K_0.25_V_2_O_5_ (0.742 nm) and KV_3_O_8_ (0.758 nm), ensuring sufficient and fast Zn^2+^ insertion/deinsertion; (iii) KVO is fabricated with one-dimensional (1D) nanowire structure, which not only facilitates direct electron transport and shortens ion diffusion pathway, but also helps to form highly porous electrode film architecture, thus increasing the interfacial contact with electrolyte. As a result, our KVO cathode exhibits good cycling stability up to thousands of times, high capacity (~ 419.4 mAh g^−1^ at 0.05 A g^−1^) and excellent rate performance. The bendable device delivers a highest energy density of 268.2 Wh kg^−1^, exceeding many previous V-based AZIBs; it also demonstrates low self-discharge rate and good temperature/pressure suitability.

## Experimental Details

### Synthesis of K_0.486_V_2_O_5_ (KVO)

Typically, 0.1818 g of V_2_O_5_ powders, 2.249 g of KI and 2.2365 g of KCl were mixed in 30 mL of deionized water and stirred for 60 min. Then, the resulting mixture solution was added into a 50 mL Teflon-lined stainless steel autoclave, which was sealed and heated in an electric oven at 200 °C for 24 h. After the reaction, the resulting precipitate was collected by centrifugation and washed with deionized water for several times. Finally, the product was dried at 80 °C in the oven for 24 h to obtain KVO [33].

### Characterization Techniques

The morphology and crystalline structure of samples were characterized using a JEOL JSM-7800F field emission scanning electron microscope (SEM) with energy dispersive X-ray spectroscopy and a JEM 2010F high-resolution transmission electron microscope (HRTEM). X-ray powder diffraction (XRD) patterns were measured on a Bruker D8 Advance diffractometer using Cu Kα radiation. Fourier transform infrared (FT-IR) spectroscopy (Nicolet Is5, ThermoFishe, USA) was performed to examine the bonding structure of KVO. X-ray photoelectron spectroscopy (XPS, Thermo Electron, VG ESCALAB 250 spectrometer) was also used to analyze the ion valence states. The ionic conductivity of the aqueous ZnCl_2_ electrolyte with different concentrations was tested on a DDS-307 conductivity meter (Shanghai Leici, China). The amount of dissolved vanadium in electrolytes was measured by a Perkin Elmer inductively coupled plasma-optical emission spectrometry (ICP-OES) Optima 730. The device thickness of the Zn//KVO batteries was measured on a digital thickness gauge (Zhejiang Syntek, China).

### Electrochemical Testing

The mass of electrode materials was measured on a microbalance with an accuracy of 0.01 mg. Cyclic voltammetry and galvanostatic charge–discharge measurements were all performed using a CS310 electrochemical workstation. Electrochemical impedance characterization was carried out on a PGSTAT100N electrochemical workstation (Autolab) to test the ionic conductivities of gel electrolyte in the frequency range between 0.01 and 10^6^ Hz at room temperature. KVO electrode was fabricated by homogenously mixing KVO, Polytetrafluoroethylene (PTFE) and acetylene black by grinding for 15 min; the homogeneous electrode material was then pressed onto Ti mesh under the pressure of 10 MPa. Full cell devices were constructed with a KVO cathode (mass loading: 2.5 mg cm^−2^; 5 and 10 mg cm^−2^ were also used for comparative study) and an excessive Zn powder anode in opposition to each other in 15 m ZnCl_2_ aqueous electrolyte; the used mass ratio of cathode to anode is 1:2.2. To assembly the quasi-solid-state device, the Zn anode and KVO cathode were firstly coated with CMC–ZnCl_2_ sol and then assembled face to face for gelation. After the CMC–ZnCl_2_ sol solidified into gel, it also acted as the separator. The sol electrolyte was prepared as follows: 0.7 g CMC and 20.5 g ZnCl_2_ were dissolved in 10 mL distilled water with vigorously stirring for 1 h at 65 °C until a uniform sol was generated.

The following equation (Eq. 1) was used to calculate Zn^2+^ ionic conductivity (*σ*) of gel electrolyte.

1 $$\sigma = d/RS$$

where *d* represents the thickness of electrolyte, *S* is the area of the electrolyte, and *R* is the ohmic resistance obtained from the impedance spectrum.

The specific capacities were calculated from galvanostatic charge/discharge curves by using Eq. 2:

2 $$Q_{spec.} = I \times t/3.6m$$

where *I* is the discharging current (A), *t* is the discharging time (s), and *m* is the mass of active materials in cathode (g). The specific energy and power densities (*E* and *P*) were calculated according to Eqs. 3 and 4, respectively.

3 $$E =\int_{0}^{\vartriangle t} IV \left( t \right){\text{d}}t/A$$

4 $$P = E/\Delta t$$

where *I* is the discharging current (A), *V*(t) is discharging voltage at *t*(*V*), d*t* is time differential and Δ*t* is the discharging time (s), *A* is the mass of KVO or device’s volume.

## Results and Discussions

### Morphology and Structure of KVO Nanowire Cathode

The KVO cathode was synthesized using a simple hydrothermal method. The experimental details are provided in the Supporting Information. XRD and the corresponding Rietveld refinement result in Fig. 2b confirm high purity of K_0.486_V_2_O_5_ (JCPDS card no. 86-0347). The element compositions and chemical state were analyzed by XPS. As shown in the survey spectrum (Fig. S1), K, V, and O elements are detected. The high-resolution V 2p_3/2_ spectrum in Fig. 2c can be divided into two peaks with binding energies located at 517.7 and 516.3 eV, attributed to V^5+^ and V^4+^, respectively [33, 34]. The bonding structure was further revealed by FT-IR spectroscopy (Fig. S2). The very weak peak at 1627 cm^−1^ is the bending vibration mode of O–H, which may be attributed to the surface-adsorbed free water in the sample. The absorption band at 994 cm^−1^ is assigned to the symmetric stretching mode (υ_s_) of V=O, and the characteristic peaks at 767 and 523 cm^−1^ correspond to the asymmetric and symmetric stretching vibrations of V–O–V bands, respectively [33]. SEM and TEM images in Figs. 2d, e and S3a, b were used to observe the size and morphology of the as-prepared KVO, where the nanowire structure (with an average width of ~ 100 nm) is distinctly confirmed. The selected area electron diffraction (SAED) pattern, presented in the inset of Fig. 2e, indicates the intrinsic single-crystal nature of KVO nanowires. A lattice fringe with a *d*-spacing of 0.195 nm is detected in the HRTEM image of KVO, corresponding to the (600) facet, as shown in Fig. 2f. Consistent with XPS analysis, energy-dispersive X-ray spectroscopy (EDS) result in Fig. S4 also confirms the nanowire component. The elemental mappings (Fig. 2g–j) further demonstrates the homogenous distribution of K in the KVO nanowire, indicating that K ions have been successfully incorporated into the layered structure.

### Electrolyte Concentration Regulation, Zn^2+^ Storage Mechanism and Performance of Zn//KVO Battery

**Fig. 3:**
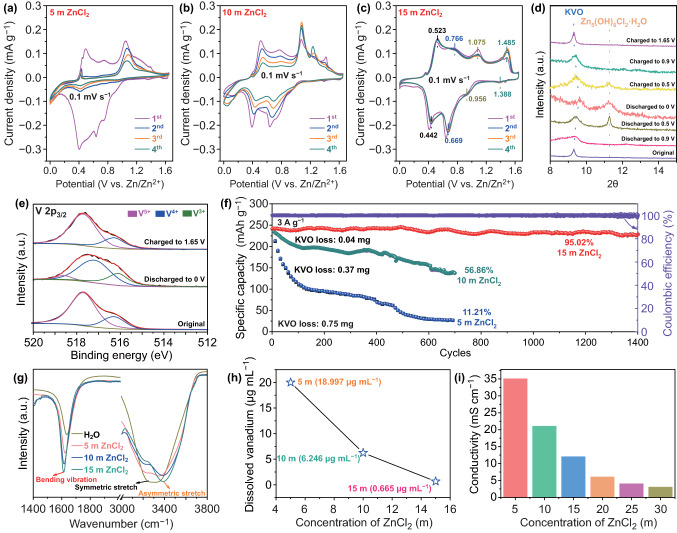
**a–c** CV curves of KVO measured at a scan rate of 0.1 mV s^−1^ in 5 m ZnCl_2_, 10 m ZnCl_2_, and 15 m ZnCl_2_ electrolyte, respectively. **d** XRD patterns of KVO and **e** high-resolution XPS spectra of V 2p at different cell states. **f** Cycle stability comparison in 5, 10, and 15 m ZnCl_2_ electrolytes at 3 A g^−1^. **g** FT-IR spectra of different electrolytes. **h** Content of dissolved vanadium in different electrolytes after KVO cycling. **i** Ionic conductivity values for the ZnCl_2_ electrolytes with different concentrations

To gain insights into the influence of ZnCl_2_ electrolyte concentration on the electrochemical stability of KVO cathode, we purposely performed the cyclic voltammetry (CV) towards our Zn//KVO battery in 5, 10, and 15 m ZnCl_2_ electrolyte at 0.1 mV s^−1^, respectively (Fig. 3a–c). Clearly, the peak intensities fade very quickly during cycling at the low concentrations and gradually stabilize with increasing the electrolyte concentration from 5–15 m; with 15 m ZnCl_2_, the 4th CV profile is almost overlapped with the first cycle. The CVs exhibit four pairs of redox peaks at ~ 0.442/0.523, 0.669/0.766, 0.956/1.075, and 1.388/1.485 V, which are attributed to the V valence state change associated with the Zn^2+^ and H^+^ insertion/extraction. To confirm the energy storage mechanism, ex-situ XRD measurements were conducted on the cathode at various discharge/charge stages, as shown in Fig. 3d. In general, no detectable phase change can be observed for the KVO cathode. During the discharge process from 1.65 to 0 V, the diffraction peak related to (001) facets at 9.3° of KVO gradually shifts toward higher angles, resulting from the decrease of interlayer spacing caused by the Zn^2+^ insertion [35] (attracting the negatively charged V–O slabs). Meanwhile, a new XRD peak at 11.2° is observed, which can be indexed to Zn_5_(OH)_8_Cl_2_·H_2_O (JCPDS No. 72-1444). The result indicates that protons are inserted into structure of KVO upon discharge and the water dissociation provides OH^−^ for the formation of this new phase. When charged to 1.65 V, the (001) peak gradually returns back to the original position with the disappearance of Zn_5_(OH)_8_Cl_2_·H_2_O signals, indicative of good Zn^2+^/H^+^ insertion/deinsertion reversibility. All these results are in good agreement with previous reports on other vanadate cathodes [18, 35]. The XPS analysis was further used to investigate the valence change of V in KVO during the discharge/charge process (Fig. 3e). As expected, with gradually discharged, the V^5+^ signal decreases, the V^4+^ signal increases and another V^3+^ component at 516.1 eV appears. Note that the V^5+^ and V^4+^ peaks shift to higher binding energies, which can be ascribed to the intercalation of Zn^2+^ ions and the simultaneous bonding rearrangements of V^5+^ and V^4+^ [21, 22, 28]. After full charging, the valence states of V return back to the original V^5+^ and V^4+^, further demonstrating a substantially reversible structure evolution.

Also, the significant difference of ZnCl_2_ concentration regulated cycling performance is evidenced by the galvanostatic charge–discharge (GCD) results at 3 A g^−1^ in Figs. 3f and S5. The Zn//KVO battery exhibits superior cycling stability up to 1400 cycles with ~ 95.02% capacity retention in 15 m ZnCl_2_ electrolyte, which is much better than that in 5 m (11.21%, 700 cycles) and 10 m ZnCl_2_ (56.86%, 700 cycles). The coulombic efficiency value also keeps at a high level of ~ 99.99% (close to ~ 100%) till the end of cycling in 15 m ZnCl_2_ electrolyte. The above results unambiguously reveal that the dissolution of KVO can be inhibited in the 15 m ZnCl_2_ electrolyte, benefited from the increased concentration features of relatively high viscosity and few “free water”. Such dissolution inhibition is also reflected by comparing the mass changes of KVO cathode before and after the cycles in three concentration electrolytes (inset of Fig. 3f; 0.75, 0.37, and 0.04 mg for 5, 10, and 15 m ZnCl_2_ electrolyte, respectively). XRD pattern of KVO cathode after 1400 charge/discharge cycles in 15 m ZnCl_2_ has also been recorded (Fig. S6). Clearly, all diffraction peaks are still in accordance with the standard pattern of pristine K_0.486_V_2_O_5_ (JCPDS No. 86-0347), proving that the crystal structure is not changed. To understand the fundamentals of electrolyte regulation strategy, we conducted FT-IR spectroscopy measurements on ZnCl_2_ electrolyte with different concentrations. As displayed in Fig. 3g, with the gradual addition of ZnCl_2_ (from 0 to 15 m), the peak of H–O–H bending vibration shifts to low wavenumber (from 1636 to 1611 cm^−1^), resulting from the increased viscosity of the electrolyte (reduced free water molecules) [32]. For the O–H stretching band, the peak related to symmetric stretch decreases from 3250 to 3200 cm^−1^ and that of asymmetric stretch increases from 3340 to 3400 cm^−1^, which clearly indicate that the hydrogen bonding network structure of water is significantly disturbed and there is strong binding of water with Zn^2+^ [32]. Exactly due to the above intrinsic features of 15 m ZnCl_2_ electrolyte, limited dissolution of vanadium from KVO cathode can be expected. This assumption was further evidenced by ICP-OES measurement toward different concentration ZnCl_2_ electrolytes after KVO cycling. As shown in Fig. 3h, the content of vanadium ions is 18.997 μg mL^−1^ in the 5 m ZnCl_2_ electrolyte. When the concentration of ZnCl_2_ reaches 15 m, very few vanadium ions (only 0.665 μg mL^−1^) were detected, demonstrating that vanadium dissolution is indeed almost prevented with increased salt concentration. It should be emphasized that such 15 m ZnCl_2_ is moderately concentrated, different from the highly concentrated WIS electrolyte (threshold value: 30 m) [32]. Apart from ensuring long cycle life of KVO, it is capable of delivering relatively high ionic conductivity of 12 mS cm^−1^. This value was much superior to that of 20–30 m higher-concentration ZnCl_2_ and was 4 times that of the 30 m ZnCl_2_ WIS electrolyte (3 mS cm^−1^), as illustrated in Fig. 3i.

**Fig. 4:**
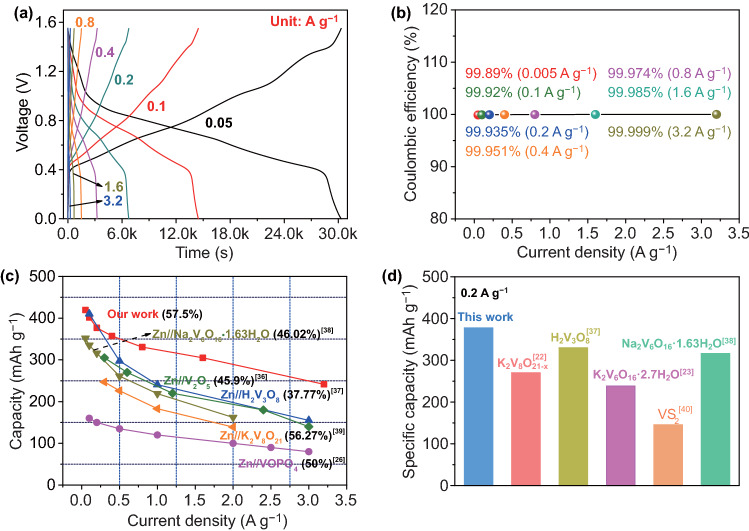
**a** GCD curves and **b** the corresponding coulombic efficiencies of KVO in optimized electrolyte of 15 m ZnCl_2_. **c** Rate performance comparison. Data from previous studies are included [26, 36–39]. **d** Specific capacity comparison at 0.2 A g^−1^. Data from previous studies are included [22, 23, 37, 38, 40]

Using the optimized 15 m ZnCl_2_ as electrolyte, our Zn//KVO battery exhibits excellent rate capability. Figure 4a illustrates the GCD profiles at various current densities of 0.05 to 3.2 A g^−1^. Several slopping plateaus are observed during charging and discharging, in good agreement with the CVs. At each current density, the coulombic efficiency approaches 100% (Fig. 4b), further indicative of the highly reversible Zn storage in the KVO nanowires. The specific capacities are calculated and plotted as a function of current rate, as displayed in Fig. 4c; rate performance of previously reported V-based cathodes are also included for comparison. At 0.05 A g^−1^, the Zn//KVO battery exhibits a maximum specific capacity of ~ 419.4 mAh g^−1^. When the current increases about 64 times to 3.2 A g^−1^, our battery can still deliver a high capacity of ~ 241.46 mAh g^−1^, which is 57.5% of the maximum capacity. Such high capacity retention within wide current density range is evidently superior to recently reported Zn//V-based batteries including Zn//V_2_O_5_ (~ 45.9%) [36], Zn//H_2_V_3_O_8_ (37.77%) [37], Zn//Na_2_V_6_O_16_·1.63H_2_O (46.02%) [38], Zn//K_2_V_8_O_21_ (56.27%) [39], and Zn//VOPO_4_ (50%) [26]. Moreover, the specific capacity of our device reaches to ~ 377.24 mAh g^−1^ at 0.2 A g^−1^ (Fig. 4d), which exceeds those of most recent AZIBs using K_2_V_8_O_21-x_ (270 mAh g^−1^) [22], H_2_V_3_O_8_ (330 mAh g^−1^) [37], K_2_V_6_O_16_·2.7H_2_O (238.4 mAh g^−1^) [23], VS_2_ (145.3 mAh g^−1^) [40], and Na_2_V_6_O_16_·1.63H_2_O (316 mAh g^−1^) [38] cathodes at the same current density. The performance enhancement should be ascribed to the aforementioned large interlayer spacing and 1D single-crystalline structure of K_0.486_V_2_O_5_.

### Electrochemical Performance of the Quasi-Solid-State Zn//KVO Battery

**Fig. 5:**
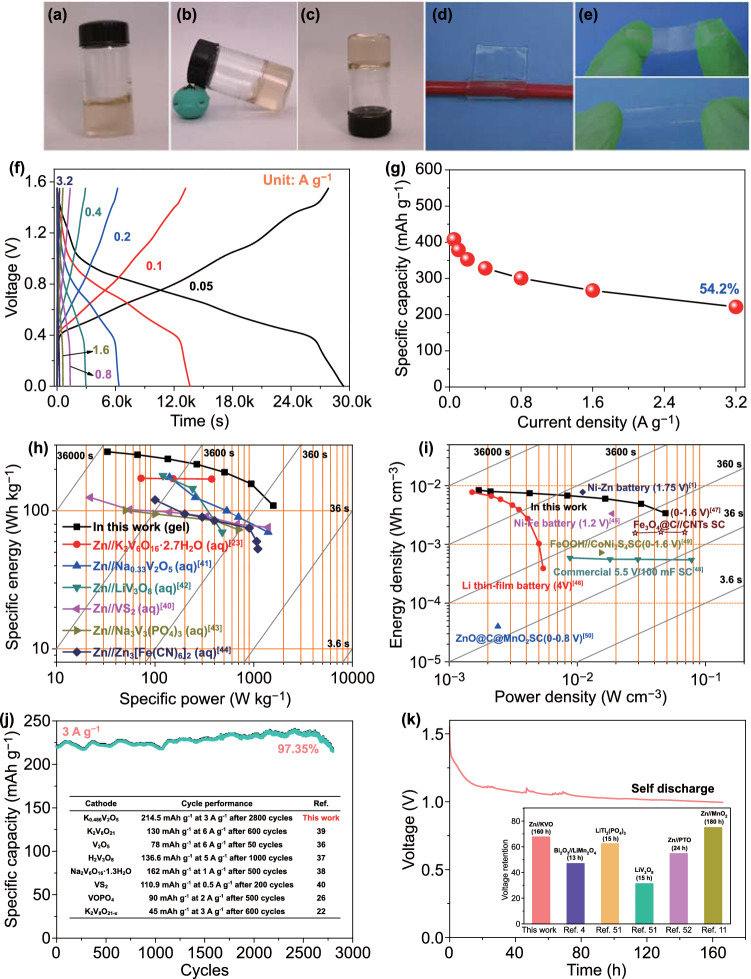
Photographs of gel electrolyte with **a–c** quasi-solid, **d** flexible and **e** stretchable characteristics. **f** Charge–discharge profiles of quasi-solid-state Zn//KVO cell. **g** Rate performance. Ragone plots of the device based on **h** cathode mass and **i** the volume of the device. Data from previous studies are included for comparison [1, 23, 40–50]. **j** Cyclic performance and the comparison with literature values (inset table) [22, 26, 36–40]. **k** Self-discharge curve and the comparison with previous batteries [3, 11, 51, 52]

Low-cost bendable energy storage devices are highly required to power future portable/wearable electronics. To further demonstrate the superiority of our Zn//KVO battery, we designed a new sodium carboxymethyl cellulose (CMC)-15 m ZnCl_2_ gel electrolyte as both the electrolyte and separator and fabricated a quasi-solid-state bendable Zn//KVO battery. The ionic conductivity of the hydrogel electrolyte was estimated as high as 10.08 mS cm^−1^ based on the impedance result in Fig. S7, which is comparable with the liquid electrolyte of 15 m ZnCl_2_. As illustrated in Fig. 5a–e, the hydrogel electrolyte is immovable and shows excellent flexibility; it can be rolled and stretched.

Figure 5f presents the GCD curves of the quasi-solid-state device at different current densities. Impressively, our device shows similar charging and discharging profiles to that with liquid aqueous electrolyte, implying that using highly conductive quasi-solid-state electrolyte does not have essential influence on the Zn^2+^ storage reaction mechanism and electron/ion transport kinetics. Good rate capability is demonstrated in Fig. 5g, which is also close to that in liquid aqueous electrolyte. The Ragone plot for our quasi-solid-state Zn//KVO device is further presented in Fig. 5h. The device is capable of delivering a maximum energy density of 268.2 Wh kg^−1^ at a power density of 32.85 W kg^−1^, much higher than that of previous aqueous Zn-V batteries such as Zn//K_2_V_6_O_16_·2.7 H_2_O (172.1 Wh kg^−1^) [23], Zn//Na_0.33_V_2_O_5_ (175 Wh kg^−1^) [41], Zn//LiV_3_O_8_ (180 Wh kg^−1^) [42], Zn//VS_2_ (125 Wh kg^−1^) [40], Zn//Na_3_V_2_(PO_4_)_3_ (101 Wh kg^−1^) [43], and Zn//Zn_3_[Fe(CN)_6_]_2_ (120 Wh kg^−1^) [44]. The gravimetric energy density remains 109.2 Wh kg^−1^ at high power density of 1578.79 W kg^−1^, indicating that our device can simultaneously exhibit high energy and power densities. To highlight the volumetric Zn^2+^ storage performance, the plot of volumetric energy density versus power density of the quasi-solid-state device is displayed in Fig. 5i. The maximum volumetric energy density of 8.38 mWh cm^−3^ is achieved, much better than those of flexible Ni//Fe batteries (3.34 mWh cm^−3^ at 18.67 mW cm^−3^) [45] and thin film Li battery [46], and comparable with previous flexible Ni//Zn batteries (7.76 mWh cm^−3^ at 11 mW cm^−3^) [1]. It is noticed that the average operating voltage (~ 0.75 V) of our device is lower than those of previous alkaline batteries (given in Fig. 5i); nevertheless, in principle, high-voltage devices of the present technology can be achieved by connecting the cells in series. Our devices also exhibit maximum volumetric power density of 48.7 mW cm^−3^, comparable to commercial 5.5 V/100 mF SC and Fe_3_O_4_@C//CNTs hybrid capacitor [47–50]. Additionally, the electrochemical stability of the quasi-solid-state device is shown in Fig. 5j. Its capacity retention is as high as ~ 97.35% even after 2800 cycles, better than using liquid 15 m ZnCl_2_ and superior to previous reported AZIBs such as Zn//H_2_V_3_O_8_ (136.6 mAh g^−1^ at 5 A g^−1^ after 1000 cycles) and Zn//K_2_V_8_O_21-x_ (45 mAh g^−1^ at 3 A g^−1^ after 600 cycles) [22, 26, 36–40] (Inset in Fig. 5j). Such further improvement in the lifetime is probably due to the additional assistance of gel electrolyte in preventing KVO from dissolution. The self-discharge performance was evaluated after being charged to 1.55 V. As shown in Fig. 5k, the device voltage remains 1.0 V (~ 64.5% of the initial voltage) after 160 h statically placed. The self-discharge time surpasses previous Bi_2_O_3_//LiMn_2_O_4_ battery (13 h; 47%), aqueous Li-ion battery anodes of LiTi_2_(PO_4_)_3_ (15 h; 62.5%) and LiV_3_O_8_ (15 h; 31.25%) as well as Zn//pyrene-4,5,9,10-tetraone (PTO) battery (24 h; 54.79%) and is comparable to Zn//MnO_2_ battery (180 h; 75%) [3, 11, 51, 52].

### Environmental Suitability of the Quasi-Solid-State Zn//KVO Battery

**Fig. 6:**
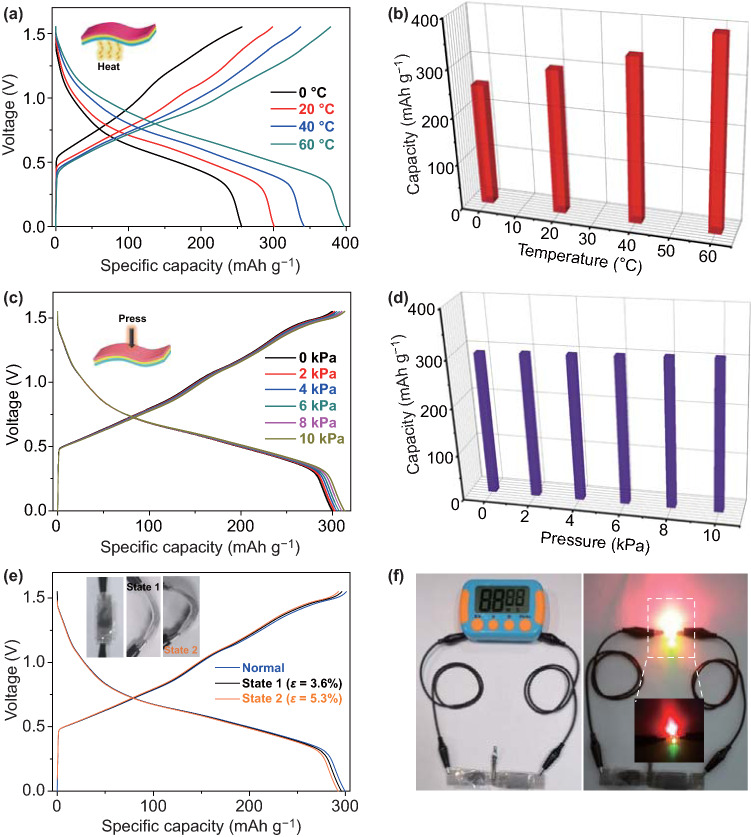
Quasi-solid-state Zn//KVO cell. **a** GCD curves at different temperatures and **b** plot of discharge capacity versus temperature. **c** GCD curves under different pressures and **d** plot of discharge capacity versus mechanical pressure. **e** GCD curves under different bending conditions (inset is the optical images of the device at the normal and 2 bendable states). **f** Optical images of a digital timer and three LED indicators lightened by the device

The quasi-solid-state Zn//KVO battery also has good environmental suitability. We performed GCD tests towards the as-fabricated quasi-solid-state device at different environmental temperatures varying from 0 to 60 °C. In Fig. 6a, b, it is shown that the device can still work well at higher temperatures, with the capacity even increased when elevating the temperature. At high temperatures, the interfacial contact and ion transfer are probably improved due to the increased wettability of the gel electrolyte, which should account for this capacity enhancement. Figure 6c, d further demonstrates the pressure resistance ability of our quasi-solid-state device. The capacity keeps ultra-stable upon being subjected to different pressures ranging from 0 to 10 kPa. It is believed that the CMC–ZnCl_2_ gel electrolyte has sufficient mechanical stiffness as in Fig. 6d, which helps to avoid the significant reduction of battery thickness and short circuit.

In addition to resist to high temperature and high pressure, our device also shows good mechanical bendability. According to previous report [53], the bendable ability/potential can be evaluated by referring to tensile strain (*ε* = $\frac{h}{2r}$,, where *h* is the cell thickness, *r* is the bending radius of curvature). The GCD voltage plateaus and capacity of the device are almost unchanged under different bending states (state 1: *ε* = 3.6%; state 2: *ε* = 5.3%), as illustrated in Fig. 6e and its inset. The tensile strain value of *ε* = 5.3% (> 5%) indicates that our device meets the bending requirement of wearable electronic products such as medical patch, wearable heater, watch belt and bendable phone [53]. To demonstrate the practical application potential, we assembled two prototype batteries in series. After full charging, the device efficiently powers a digital timer (1.5 V) and can simultaneously light up red (1.8 V, 0.036 W), yellow (2.1 V, 0.042 W), and green (2.3 V, 0.046 W) LED indicators brightly (Fig. 6f).

It should be emphasized that in real applications, batteries are generally not discharged to 0 V. From either the CV or GCD profiles, it can be seen that the majority of the capacity of our device is released above ~ 0.3 V. Thus, in principle, the device can also be used with the cut-off voltage of 0.3 V if employed for real applications.

## Electrochemical Performance of the Aqueous Zn//KVO Battery based on High KVO Mass Loading

**Fig. 7:**
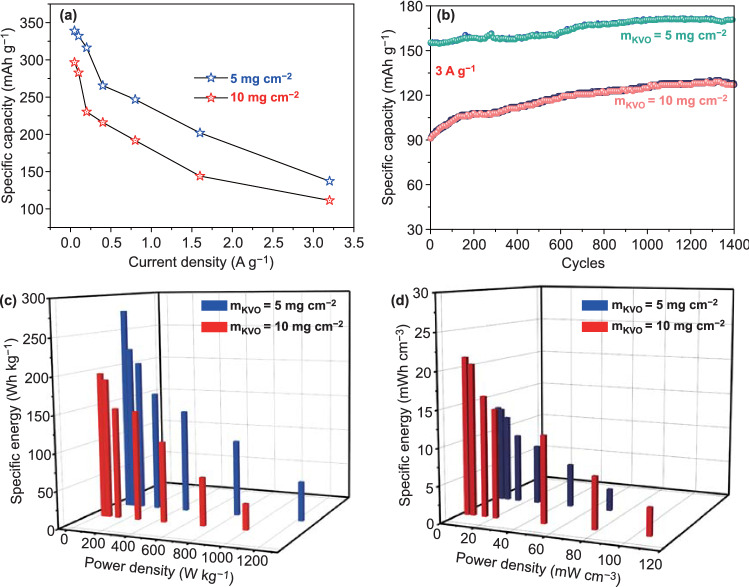
High mass loading Zn//KVO cell:** a** Rate performance. **b** cycling performance. Energy densities and power densities of the cell based on **c** KVO mass and **d** the volume of the device

At last, the electrochemical performance of aqueous Zn//KVO batteries in 15 m ZnCl_2_ based on high KVO mass loading (5 and 10 mg cm^−2^) was evaluated. GCD curves of the devices at different current densities are shown in Fig. S8a, b. The specific capacity was calculated and displayed in Fig. 7a. When operated at 0.05 A g^−1^, the Zn//KVO battery exhibits a maximum specific capacity of ~ 339 mAh g^−1^ with 5 mg cm^−2^ loading and ~ 296.64 mAh g^−1^ with 10 mg cm^−2^ loading. When the current density is increased 64 times from 0.05 to 3.2 A g^−1^, 40.47% (5 mg cm^−2^), and 37.5% (10 mg cm^−2^) of the initial capacity can be maintained, indicating that the devices with high KVO mass loading still have good rate capability. The long cycling stability is also achieved (Fig. 7b). After 1400 cycles at 3 A g^−1^, the discharge capacities of Zn//KVO batteries are ~ 171 mAh g^−1^ (5 mg cm^−2^) and ~ 128 mAh g^−1^ (10 mg cm^−2^). The continuing increase in the capacity during the cycling process is probably due to the gradual wetting of the electrode with concentrated electrolyte. Although the gravimetric energy densities (Max. 227.7 Wh kg^−1^ for 5 mg cm^−2^ loading, and 196.65 Wh kg^−1^ for 10 mg cm^−2^ loading) of the devices are slightly inferior to that with low mass loading, the volumetric energy densities are higher, which can reach the highest values of 21.4 mWh cm^−3^ (10 mg cm^−2^) and 13.2 mWh cm^−3^ (5 mg cm^−2^), as illustrated in Fig. 7c, d.

## Conclusions

In summary, we have demonstrated that the cycling stability of KVO in AZIBs can be remarkably enhanced by regulating the concentration of ZnCl_2_ electrolyte. The best cyclability can be achieved with the optimized 15 m ZnCl_2_, which avoids the use of the 30 m “WIS” electrolyte (ultrahigh viscosity and low conductivity) and maintains high ionic conductivity to realize high capacity and excellent rate performance. Furthermore, a quasi-solid-state Zn//KVO battery is also established by firstly utilizing a novel CMC-moderate concentration ZnCl_2_ hydrogel electrolyte. Benefiting from large interlayer spacing of KVO and the unique moderately concentrated ZnCl_2_-based gel electrolyte, the Zn//KVO battery can simultaneously achieve high energy and power densities as well as longer cycle life of 2800 times. Such battery devices can also well operate under elevated temperature, high mechanical pressure and various bending conditions. Our work addresses the dissolution issue of V-based cathodes and offers a smart strategy to enable other families of soluble materials for high-stability aqueous batteries.

## Electronic supplementary material

The full XPS spectrum of KVO; FT-IR analysis on KVO nanowire cathode; SEM images and EDS result of KVO nanowire cathode; Cycling comparison of KVO in 5, 10, and 15 m ZnCl_2_ electrolytes at 3 A g^−1^; XRD pattern of the cycled KVO in 15 m ZnCl_2_ electrolyte; AC impedance spectrum of CMC–ZnCl_2_ gel electrolyte at room temperature; Charge–discharge profiles of aqueous Zn//KVO cell based on high KVO mass loading.

Below is the link to the electronic supplementary material.

Supplementary file 1 (PDF 308 kb)
