# Supplementary material for: Electrolyte Concentration Regulation Boosting Zinc Storage Stability of High-Capacity K0.486V2O5 Cathode for Bendable Quasi-Solid-State Zinc Ion Batteries
Source: Nanomicro Lett. 2021 Jan 4;13:34. doi: 10.1007/s40820-020-00554-7 (PMC8187517; doi:10.1007/s40820-020-00554-7)
Supplement: Supplementary file 1 — (PDF 308 kb) [file 40820_2020_554_MOESM1_ESM.pdf]

Supporting Information for

Electrolyte Concentration Regulation Boosting Zinc Storage Stability of High-Capacity $K_{0.486}V_2O_5$ Cathode for Bendable Quasi-Solid-State Zinc Ion Batteries

Linpo Li[1, 2], Shuailei Liu[2], Wencong Liu[2], Deliang Ba[1, 2], Wenyi Liu[2], Qiuyue Gui[2], Yao Chen[2], Zuoqi Hu[1], Yuanyuan Li[1, *], Jinping Liu[2, 3, *]

[1]School of Optical and Electronic Information, Huazhong University of Science and Technology, Wuhan 430074, People's Republic of China

School of Chemistry, Chemical Engineering and Life Science, and State Key Laboratory of Advanced Technology for Materials Synthesis and Processing, Wuhan University of Technology, Wuhan 430070, People's Republic of China

[3]Key Laboratory for Photonic and Electronic Bandgap Materials, Ministry of Education, School of Physics and Electronic Engineering, Harbin Normal University, Harbin 150025, People's Republic of China

*Corresponding authors. E-mail: liyynano@hust.edu.cn (Y. Y. Li); liujp@whut.edu.cn (J. P. Liu)

Supplementary Figures

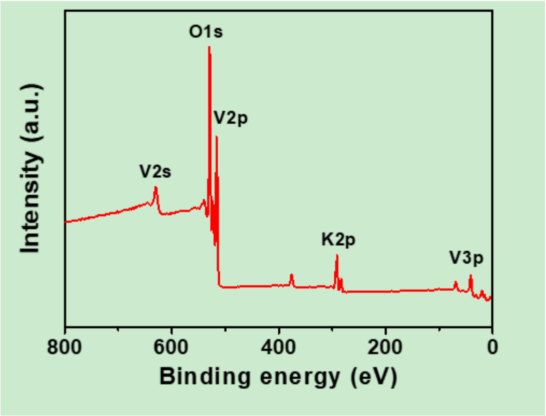

Fig. S1 Full XPS spectrum of KVO

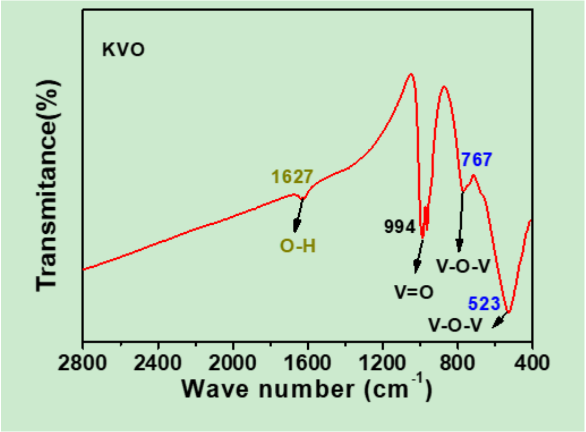

Fig. S2 FT-IR analysis on KVO nanowire cathode

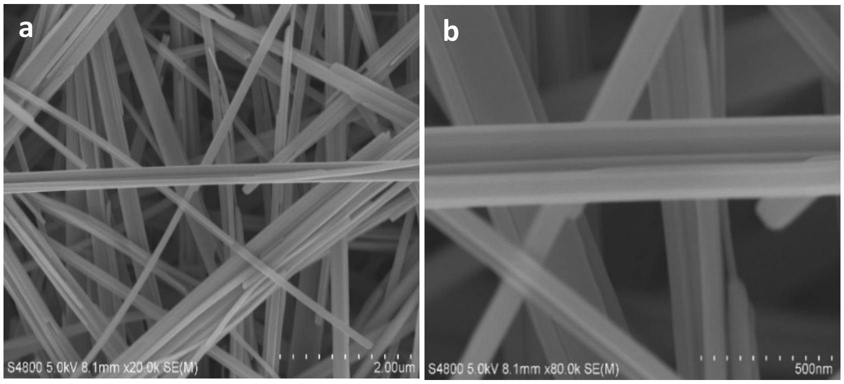

Fig. S3 SEM images of KVO nanowire cathode

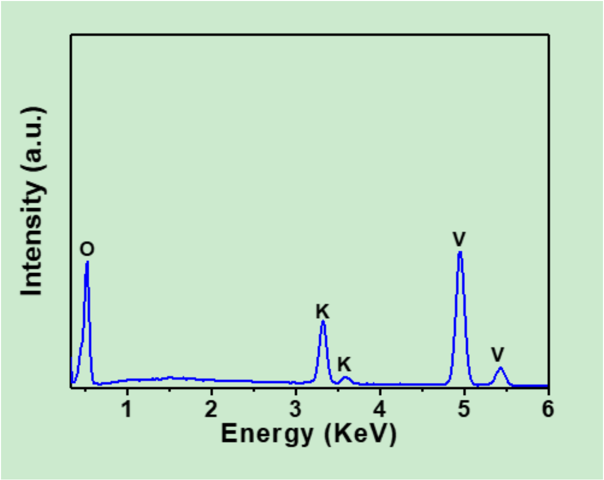

Fig. S4 EDS result of KVO nanowire cathode

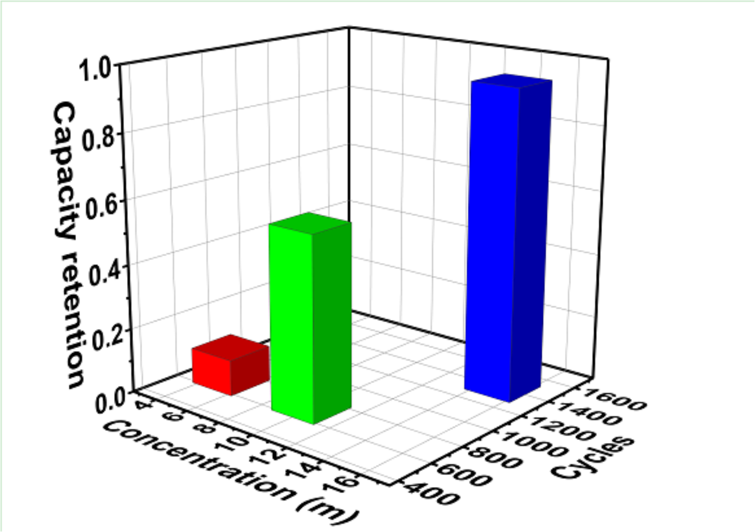

Fig. S5 Cycling comparison of KVO in 5, 10, and 15 m $ZnCl_2$ electrolytes at 3 A g^{-1}

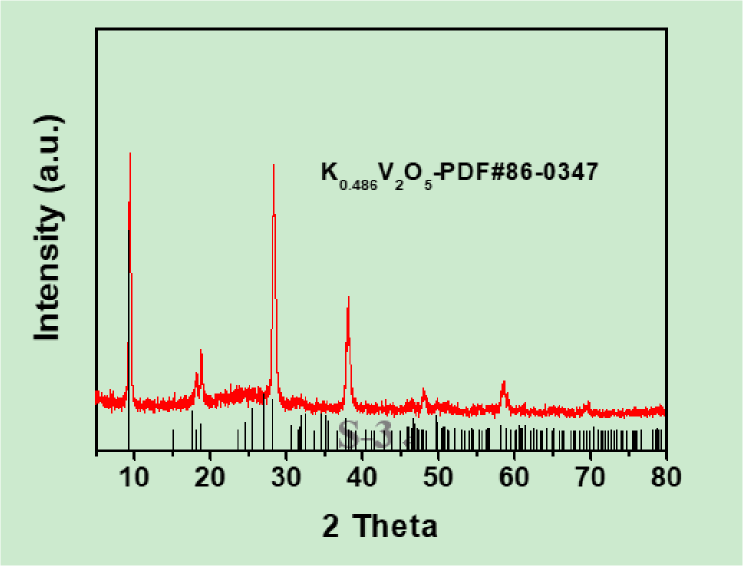

Fig. S6 XRD pattern of the cycled KVO in 15 m $ZnCl_2$ electrolyte

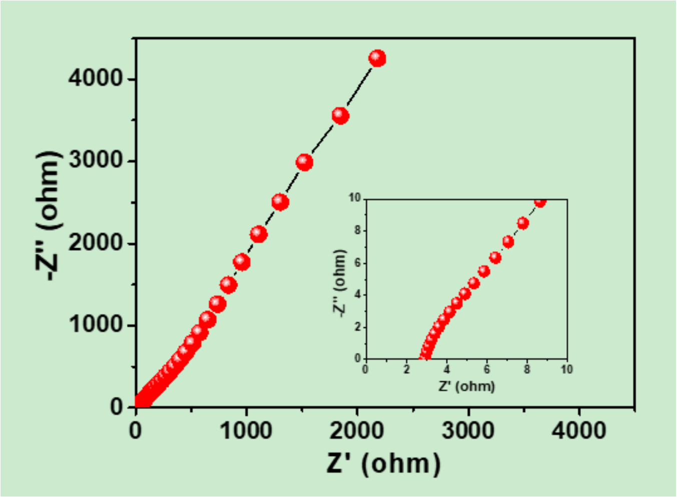

Fig. S7 AC impedance spectrum of CMC-$ZnCl_2$ gel electrolyte at room temperature

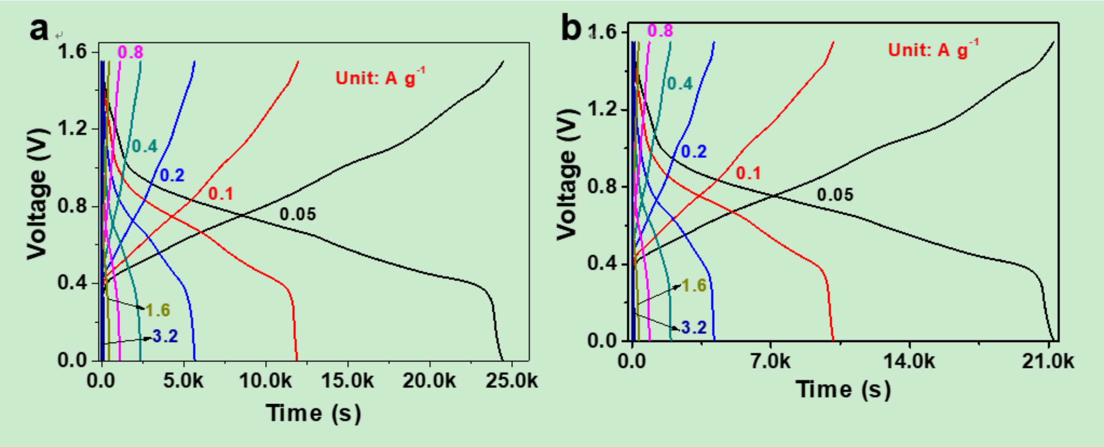

Fig. S8 Charge-discharge profiles of aqueous Zn//KVO cell based on high KVO mass loading (**a**: 5 mg cm^{-2}; **b**: 10 mg cm^{-2})
